# Action of 3-Hydroxy-3-Methylglutaryl-CoA Reductase Inhibitors on ABCA-1 protein (ATP-Binding Cassette Transporter-1) in endothelial cells stimulated with uremic serum

**DOI:** 10.1186/s12944-024-02420-6

**Published:** 2025-03-19

**Authors:** Silmara de Melo Carmona, Daniele Pereira Jardim, Maria Aparecida Dalboni, Renata Nakamichi, Mariana Kuniyoshi, Mauro Marrocos, Beata Marie Redublo Quinto, Marcelo Costa Batista

**Affiliations:** 1 Federal University of São Paulo, Rua Pedro de Toledo, 669, 10th floor, CEP 04039-032, São Paulo, SP Brazil; 2 Albert Einstein Israelite Hospital, São Paulo, Brazil; 3 Nove de Julho University, São Paulo, Brazil

**Keywords:** 3-Hydroxy-3-Methylglutaryl-CoA Reductase Inhibitor, ABCA, 1, Endothelium, Uremia

## Abstract

The high incidence of cardiovascular events in chronic kidney disease (CKD) exhibits an epidemic character, affecting patients in predialysis, hemodialysis, and post-transplant stages, accounting for approximately 50% of deaths, with a mortality rate around 9% per year. Statins are primarily used in the treatment of dyslipidemia and possess pleiotropic effects that are not yet fully understood. Moreover, there is insufficient evidence to support their use in attenuating cardiovascular outcomes or their potential role in modulating the ABCA-1 transporter in CKD. Thus, the aim of this study was to investigate the effect of statins on ABCA-1 modulation in cultures of human umbilical vein endothelial cells (HUVEC) incubated with uremic serum from CKD patients. The results demonstrate that statins influence the inflammatory response of HUVEC exposed to a uremic environment by reducing TNF-α secretion compared to baseline levels. Furthermore, the uremic environment was found to decrease the expression of LxR-β and RxR-α, leading to a consequent reduction in ABCA-1 expression in HUVEC. Cells pre-treated with simvastatin exhibited increased expression of ABCA-1, LxR-β, and RxR-α, along with a significant increase in the transcription of ABCA-1, LXR-β, and RxR-α, indicating that statins may exert a positive modulation on LxR-β and RxR-α receptors, activating ABCA-1 transcription.

**Graphical Abstract:**

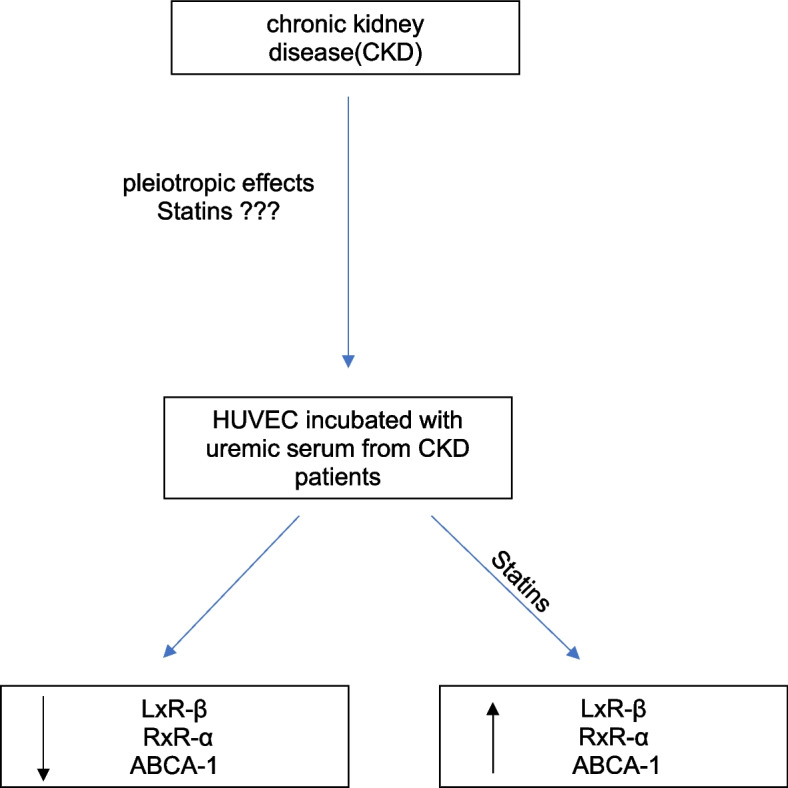

**Supplementary Information:**

The online version contains supplementary material available at 10.1186/s12944-024-02420-6.

## Highlights

• The uremic environment reduces the expression of LxRα, LxRβ and RxRα

• Decreased gene expression of LxRs and RxRα receptors in uremic environment was associated with the diminished expression of ABCA-1

• Statins act on the inflammatory response of HUVEC cells exposed to the uremic environment by decreasing TNF-α secretion when compared to basal secretion

• Uremic environment reduces the expression of LxR-β and RxR-α leading to a consequent decrease in ABCA-1 expression in HUVEC

• Statins may exert positive modulation on the LxR-β and RxR-α receptors for ABCA-1 transcription activation

**Supplementary Information:**

The online version contains supplementary material available at 10.1186/s12944-024-02420-6.

## Introduction

Abnormalities in lipid metabolism are associated with the progression of kidney disease and are prevalent at all stages of chronic kidney disease (CKD). These abnormalities can contribute to the development of atherosclerosis, which is closely linked to cardiovascular events and increased inflammatory responses due to endothelial injury. Reverse cholesterol transport (RCT) is a mechanism responsible for removing excess cholesterol from extrahepatic tissues and is fundamental for vascular protection, thereby preventing atherosclerosis.

A key component of RCT, specifically for cholesterol efflux, is the cytoplasmic protein ATP-binding cassette transporter 1 (ABCA1), a 264 kDa protein mapped to chromosome 9q32. The atheroprotective action of ABCA1 has been demonstrated in models of both overexpression and deficiency of this transporter in liver cells [1] and in transgenic rodent macrophages [2–4].

The main transcription factors involved in the upregulation of ABCA1 transcription are the liver X receptors α and β (LxRα, β) and the retinoid X receptors (RxR). The LxR class of receptors serves as a primary regulator of lipid homeostasis [5]. Activation of LxR and RxR dimers occurs physiologically through oxysterols, retinoids, or their agonists, which stimulate the genetic transcription of ABCA1 via the DR4 promoter element, thereby increasing cholesterol efflux [5–9].

Cytokines such as tumor necrosis factor α (TNF-α), interleukin-1β (IL-1β), and interferon-γ (IFN-γ) [10] can negatively regulate LxR-mediated ABCA1 transcription and its protein expression [11, 12]. Given that CKD patients often experience a state of microinflammation primarily due to uremic toxins, this inflammatory response, combined with the downregulation of ABCA1, may exacerbate endothelial injury.

The anti-inflammatory and antioxidant roles of ABCA1 were suggested in a study involving human coronary artery endothelial cells incubated with the 5A peptide, an ApoA-I mimetic that facilitates cholesterol removal from lipid microdomains formed by ABCA1. When stimulated with TNF-α, this treatment resulted in significant inhibition of adhesion molecule expression, reactive oxygen species (ROS), and the activation pathway of nuclear factor kappa B (NF-κB) [13]. In vivo studies utilizing acute inflammation models demonstrated that administration of the 5A peptide inhibited adhesion molecules and ROS, indicating anti-inflammatory and antioxidant effects mediated by ABCA1 [13]. Additionally, Franzin et al. found that THP-1 macrophage cells treated with phorbol 12-myristate 13-acetate, an inducer of macrophage differentiation [14], exhibited increased ABCA1 expression when exposed to uremic conditions compared to cells exposed to normal serum [15].

ABCA1 plays a crucial role in endothelial function by mediating reverse cholesterol transport, thereby providing vascular protection. Dyslipidemia and increased oxidative stress in CKD patients contribute to endothelial dysfunction, promoting atherosclerosis. In addition to its role in cholesterol efflux, ABCA1 has anti-inflammatory and antioxidant effects, limiting the production of ROS and reducing the expression of endothelial adhesion molecules. This suggests that modulating ABCA1 could be a therapeutic strategy to mitigate endothelial damage in CKD patients, particularly in the context of chronic inflammation [16].

There is growing interest in identifying effective therapeutic strategies to mitigate endothelial dysfunction in uremia and, consequently, reduce the burden of atherosclerotic disease in CKD patients. HMG-CoA reductase inhibitors, known as statins, are widely used in the treatment of dyslipidemia and have been shown to reduce the incidence of cardiovascular diseases. Primary and secondary prevention studies have demonstrated that statins inhibit the secretion of matrix metalloproteinases from macrophages, negatively regulate the expression of scavenger receptor class A, improve endothelial dysfunction, and stabilize atherosclerotic plaques [17]. Studies have also shown that, in CKD patients, statins have beneficial effects on inflammation, particularly in reducing inflammatory cytokine production and improving endothelial function, making them promising in the context of CKD [16]. Dyslipidemia is prevalent in patients with CKD and is associated with a significantly increased cardiovascular risk [16]. This lipid disorder is often characterized by elevated triglyceride levels, low high-density lipoprotein (HDL) levels, and increased low-density lipoprotein (LDL) levels [1]. These lipid abnormalities contribute substantially to the progression of atherosclerosis and endothelial dysfunction. One of the mechanisms underlying dyslipidemia in CKD is the decreased expression of ABCA1, which leads to reduced cholesterol efflux and lipid accumulation in endothelial cells, exacerbating the risk of cardiovascular diseases. The dysfunction in cholesterol transport, mediated by ABCA1, compromises the body's ability to remove cholesterol from arterial walls, increasing the propensity for the formation of atherosclerotic plaques [18, 19].

At the cellular level, our group has previously demonstrated the anti-inflammatory effects of statins on monocytes isolated from the peripheral blood of patients hospitalized in the Intensive Care Unit [20].

Thus, this study investigates whether the pleiotropic actions of statins exert beneficial effects on vascular protection through potential modulation of ABCA1 transcription in the context of uremia.

## Methods

### Materials and methods

#### Materials

Culture medium and reagents were purchased from Gibco (USA). LB agar medium and other reagents were obtained from Sigma-Aldrich. Protease inhibitors, the SCR023 kit, TRITC and FITC fluorophores, and primary monoclonal and secondary antibodies were purchased from Merck Millipore Corporation (USA). Formaldehyde solution was sourced from Merck (USA), while glycerol and DMSO were acquired from Labsynth (Brazil). APC and PE fluorophores were obtained from BD (Becton Dickinson, USA). ECL Western Blotting Substrate and Revertaid H Minus First Strand cDNA Synthesis Kit were sourced from Fermentas, Thermo Scientific (USA). The Interleukin 10 and TNF-α ELISA kits were obtained from R&D Systems (USA), while the One-Shot Top 10 Competent Cells kit was sourced from Invitrogen (USA). Human cDNA ORF Clone plasmids containing tagged GFP (Green Fluorescent Protein) and the respective ABCA-1, LxR-β, and RxR-α sequences were purchased from Origene (USA). RNA extraction was performed using the QuantiTec kit, and transfection was carried out using the Attractene kit; plasmid DNA was extracted using the QIAprep Spin kit from Qiagen (Germany), while protein quantification was conducted using a colorimetric kit from Bio-Rad (USA). Ammonium persulfate and acrylamide solutions were purchased from BioAgency (Brazil).

### Collection of uremic serum

Serum samples were collected from patients undergoing Renal Replacement Therapy (RRT) at the Kidney and Hypertension Hospital (Oswaldo Ramos Foundation / Federal University of São Paulo—UNIFESP), before the dialysis session to obtain uremic serum. Samples were pooled by adding 5 mL aliquots of serum from each of the 202 patients into a sterile conical tube, followed by homogenization and storage at −20ºC for subsequent cell incubation. This study was approved by the institutional review board, and the need for consent was waived for the retrospective analysis of anonymized data. The medical ethics committee of UNIFESP approved this study under institutional registration number 616429.

### Cell culture

Human umbilical vein endothelial cells (HUVEC) were obtained from the Cell Signaling Laboratory of the UNIFESP Cellular and Molecular Therapy Center (CTCMol-UNIFESP). The cells were thawed in 25 cm3 flasks (TPP—Techno Plastic Products, Switzerland), containing RPMI culture medium (Roswell Park Memorial Institute medium, Gibco, USA), supplemented with 10% fetal bovine serum (Gibco, USA), and 5% antibiotics (Penicillin and Streptomycin, Gibco, USA). The cells were maintained in an incubator at 37 °C with 5% CO₂ saturation. The protocol for culturing the cells followed the methodology described by Urbaczek et al. [21] [21]. Upon reaching 90% confluence, the culture medium was aspirated. The cells were washed three times with phosphate-buffered saline (PBS) and then incubated at 37 °C for 90 s with 500 µL of 0.25% trypsin (Gibco, USA). After this period, cells were centrifuged to remove trypsin, 1 mL of RPMI medium was added, homogenized, and sub cultured for further experiments. The cells were used between passages 10 and 15 to ensure their optimal growth and viability.

### Cell characterization

HUVEC cells were characterized through immunofluorescence and flow cytometry.

#### Immunofluorescence

The SCR023 kit (Millipore Corporation, USA) was used for the characterization of endothelial cells, employing monoclonal antibodies against CD31 (MABF2034, Merck Millipore), CD146 (ABT1488, Merck Millipore), vWF (MABT856, Merck Millipore), VCAM (MAB2143, Merck Millipore), and ICAM (AB1379, Merck Millipore). Anti-rabbit secondary antibodies conjugated to the fluorophore FITC (Fluorescein-5-isothiocyanate, Millipore Corporation, USA) were utilized for the von Willebrand factor (vWF) marker. A previously established protocol was followed, and observations were made under a fluorescence microscope. Characterization results indicated that HUVEC cells were positive for the specific marker VCAM and the constitutive markers CD146, CD31, and vWF.

#### Flow cytometry

Primary antibodies from the SCR023 kit (Millipore® Corporation, USA) were employed to analyze the expression of CD31, CD146, vWF, VCAM, and ICAM markers. HUVEC cells were subcultured in three 25 cm3 flasks. Two flasks were stimulated with 100 ng of TNF-α, according to the kit manufacturer’s instructions, and incubated for two hours at 37ºC to assess the expression of ICAM and VCAM. Secondary antibodies conjugated to APC and PE were added and incubated in the dark for 30 min. Fluorescence from CD31, CD146, vWF, VCAM, and ICAM on HUVEC was detected by flow cytometry (FACScanto analyzer; Becton Dickinson Immunocytometry Systems) and expressed as mean fluorescence intensity (MFI).

### Pretreatment with Simvastatin and Incubation with uremic serum

Human umbilical vein endothelial cells (HUVEC) were cultured in RPMI medium (Gibco, USA) supplemented with 10% fetal bovine serum (FBS, Gibco, USA) and 5% antibiotics (Penicillin and Streptomycin, Gibco, USA) at 37 °C in a 5% CO₂ atmosphere. The cells were divided into the following experimental groups:

1. Control group: Cells incubated with 45% culture medium supplemented with 10% FBS and 45% serum from healthy individuals.
2. Uremic group: Cells incubated with a mixture of 45% culture medium containing 10% FBS and 45% uremic serum obtained from patients with uremia. This allowed the cells to be exposed to uremic conditions while maintaining the necessary nutrients and growth factors from FBS.
3. Simvastatin group: Cells incubated with 45% culture medium supplemented with 45% serum from healthy individuals supplemented with 10% FBS and pretreated with 10 µM simvastatin for 24 h.
4. Simvastatin + Uremia group: Cells pretreated with 10 µM simvastatin for 24 h and subsequently incubated with a mixture of 45% culture medium containing 10% FBS and 45% uremic serum obtained from patients with uremia, with an additional 10 µM simvastatin. All groups were incubated for 24 h at 37ºC under optimal humidity and CO₂ conditions. After incubation, the supernatant from each group was collected in 1.5 mL microtubes and stored at −80ºC. The cells were lysed, and RNA was extracted, quantified, and stored at −80ºC.

### Expression of LxRα, LxRβ, RxRα, and ABCA1 by Real-Time PCR

#### RNA and cDNA Extraction

Total RNA was extracted from the HUVEC culture following the manufacturer's instructions using the QuantiTec kit (Qiagen, Germany). The precipitate obtained was eluted in RNase-free distilled water and quantified in ng/mL using a Nanodrop 1000 (Uniscience, USA), and stored at −80ºC. cDNA was prepared from 1 µg of total RNA using the Revertaid H Minus First Strand cDNA Synthesis Kit (Fermentas, Thermo Scientific, USA).

#### Real-Time PCR

Quantitative real-time PCR was conducted using a 7500 Real-Time PCR System thermocycler (Applied Biosystems, UK). In addition to deoxynucleotides, specific buffer, and the fluorescent dye SybrGreen, an aliquot of cDNA was combined with specific primers corresponding to the genes of interest. Serial dilutions of control samples were analyzed simultaneously to establish a standard curve. Primers for the proteins of interest were designed based on gene sequences obtained from GenBank and were synthesized by Integrated DNA Technologies (IDT). The primer sequences used are detailed in Table 1 (Supporting Information). The specificity of the generated products was confirmed by analyzing their melting curves. Products were quantified and compared to controls based on the number of cycles required to achieve a specific fluorescence value during the reaction's log-linear phase. Relative gene expression was quantified using the method as described by Livak and Schmittgen (2001) [22].The cycle threshold (Ct) values of the genes of interest were normalized to reference genes, and the data were analyzed using the 2^(-ΔΔCt) formula to calculate relative expression levels. Statistical analysis was performed in triplicate, and expression values were adjusted relative to the control for each experiment. Reactions with housekeeping primers (HPRT) were routinely performed to normalize all values obtained for the genes of interest.

**Table 1:** Demographic and Laboratory Data of Patients at Stage 5 CKD-EPI

Patient Characteristics	Value (Mean ± SD)
Age	68.4 ± 12.3 years
Gender	60% male, 40% female
Serum Creatinine (mg/dL)	8.1 ± 1.5
Blood Urea Nitrogen (mg/dL)	55.3 ± 12.7
Hemoglobin (g/dL)	9.2 ± 1.4
eGFR (mL/min/1.73 m^2^)	13.5 ± 3.2
Albumin (g/dL)	3.0 ± 0.5
C-reactive protein (mg/L)	6.7 ± 2.3
Total Cholesterol (mg/dL)	187.6 ± 28.9
Triglycerides (mg/dL)	240.4 ± 45.6

### Detection of Interleukin 10 and Tumor Necrosis Factor-α

The levels of Interleukin 10 and TNF-α in the cell supernatant were measured using enzyme immunoassay, employing the ELISA kit (R&D Systems, USA) and following the manufacturer's recommendations. Absorbance readings were performed in a spectrophotometer at a wavelength of 450 nm. The detection limits were 3.9 pg/mL for IL-10 and 0.191 pg/mL for TNF-α.

### Transfection of HUVEC Cells for Analysis of Promoter Activation Mediated by LxR and RxR-Recruiter Gene

#### Bacterial transformation

The One-Shot Top 10 Competent Cells kit (Invitrogen, USA) containing *Escherichia coli (E. coli)* was used for transformation. Human cDNA ORF Clone plasmids, containing tagged GFP (Green Fluorescent Protein) along with the respective ABCA-1, LxR-β, and RxR-α sequences, were utilized. A vial containing 50 µL of E. coli bacteria was thawed in an ice bath. Following thawing, 1 µL of dimethyl sulfoxide and 200 ng of plasmid were added. The thermal shock procedure involved initial incubation on ice for 30 min, followed by 1 min and 30 s in a wet bath at 42ºC, concluding with 2 min on ice. After the thermal shock, 1 mL of SOC medium (provided by the kit) was added, and the mixture was incubated at 37ºC with shaking at 225 rpm for 1 h and 30 min. The bacteria were then plated on LB Agar medium (Luria Bertani) (Sigma-Aldrich, USA) supplemented with 100 µg/mL ampicillin (Gibco, USA) and incubated for 24 h at 37ºC with 5% CO₂ saturation. After colony growth, bacteria were cultured in LB Broth medium supplemented with 100 µg/mL ampicillin and incubated at 37ºC with shaking at 120 rpm for 24 h. Plasmid DNA was then extracted and purified using the QIAprep Spin kit (Qiagen, Germany), quantified using a Nanodrop, and stored at −20ºC.

#### Transfection of HUVEC Cells

HUVEC cells were plated at a density of 1 × 104 in 12-well plates, organized according to the established groups, totaling n = 6 in each group. For transfection, a lipofection solution was prepared. For each sample, 400 ng of plasmid, 1.5 µL of Attractene transfection reagent (Qiagen, Germany), and culture medium without antibiotics and supplements were combined in a conical tube to achieve a final volume of 60 µL. The mixture was centrifuged for 30 s in a microcentrifuge and incubated at room temperature for 15 min. Following incubation, the solution was added dropwise into the wells of all experimental groups. A supplemented culture medium was then added to the wells, and cells were incubated for 24 h at 37ºC. After this period, groups 3 and 4 were pretreated with simvastatin for 24 h. Subsequently, groups 1, 2, and 4 were stimulated with serum from healthy and uremic individuals, remaining incubated for another 24 h at 37ºC for subsequent analysis of the reporter gene.

#### Analysis of RxRα, LxRβ, and ABCA-1 Reporter Gene

Following transfection, HUVEC cells were pretreated with 10 µM simvastatin and stimulated with healthy and uremic serum. The cells were washed with PBS, trypsinized, transferred to 12 × 75 mm cytometry tubes, and centrifuged at 2,500 rpm for 5 min. The supernatant was discarded, and the cells were resuspended in 200 µL of PBS. Fluorescence was measured using the FACS Canto flow cytometer.

### Expression of LxR, RxR, and ABCA1 by Western Blot

Total protein was extracted and quantified from HUVEC cells [23]. A total of 20 μg of protein was separated by SDS-PAGE and transferred onto nitrocellulose membranes. After blocking, membranes were probed with primary antibodies: mouse monoclonal anti-LxR, rabbit polyclonal anti-RxR, and mouse monoclonal anti-ABCA-1 α, as well as mouse anti-actin (Sigma Aldrich, St. Louis, MO, USA). Membranes were subsequently treated with peroxidase-conjugated goat anti-mouse or anti-rabbit IgG (Millipore, St. Charles, MO, USA). Specific proteins were visualized following incubation with the chemiluminescent HRP substrate (Immobilon Western, Millipore, St. Charles, MO, USA). The analysis of the Western blot bands was performed using densitometry with ImageJ software. Western blot images were imported into ImageJ, and the intensity of the bands was quantified based on relative density. Corresponding graphs were generated from the densitometry data to facilitate the interpretation and comparison of the results. It is important to note that these data represent a single sample per condition, as additional replicates were not feasible due to time and resource limitations. Further experiments with independent replicates are necessary to confirm the reliability of these findings.

### Statistical analyses

Statistical analyses were performed using SPSS software version 20.0. The results for each group were expressed as mean ± standard deviation. We applied the Kolmogorov–Smirnov test to assess the normality of the data before performing parametric or non-parametric analyses. To test the statistical significance of the differences between the analyzed variables across the studied groups, analysis of variance (ANOVA) was employed, complemented by the Bonferroni test. Spearman's coefficient was utilized to analyze the correlation between variables. A significance level of *p* < 0.05 was established for all tests.

## Results

### Demographic and laboratory data of patients at stage 5 CKD-EPI

The serum samples used for incubating HUVECs were pooled from patients diagnosed with Stage 5 of CKD according to the CKD-EPI classification. The demographic and laboratory characteristics of these patients are summarized in Table 1. These characteristics include relevant clinical parameters such as age, gender, serum creatinine levels, eGFR, and other laboratory markers that were important for the study.

### Simvastatin decreases TNF-α secretion and increases IL-10 secretion in HUVEC exposed to uremic serum

**Fig. 1:**
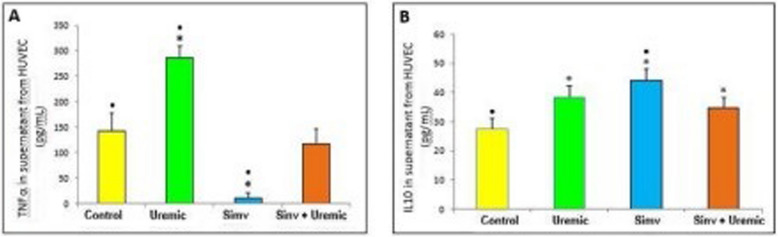
Secretion of **A**) TNF-α and **B**) IL-10 in HUVEC incubated with serum from control, uremic serum, pretretead with simvastatin and pretretead and subjected to an insult with uremic serum for 24 hours (*n*=12). *p*≤0.05 (* versus control, * versus simv + uremic), *N*=12

The incubation of HUVEC with uremic serum significantly increased TNF-α secretion compared to the group incubated with healthy serum (286.55 ± 24.07 pg/mL *versus* 140.85 ± 36.33 pg/mL, *P* < *0.05*). Cells treated with simvastatin and exposed to uremic serum showed significantly lower TNF-α secretion compared to cells exposed only to uremic serum (116.86 ± 30.00 pg/mL *versus* 286.55 ± 24.07 pg/mL, *P* < *0.05*) (Fig. 1a).

Cells pretreated with simvastatin for 24 h had lower TNF-α secretion compared to the other groups studied (simvastatin: 9.26 ± 10.03 pg/mL *versus* healthy: 140.85 ± 36.33 pg/mL; uremic: 286.55 ± 24.07 pg/mL; simvastatin + uremic: 116.86 ± 30.00 pg/mL, *P* < *0.05*).

The secretion of IL-10 by HUVEC was increased in all evaluated groups compared to the control group (uremic: 38.50 ± 3.70 pg/mL; simvastatin: 44.19 ± 3.90 pg/mL; simvastatin + uremic: 34.90 ± 3.31 pg/mL *versus* control: 27.65 ± 3.65 pg/mL, *P* < *0.05*) (Fig. 1b).

### Simvastatin increases LxRα, LxRβ, RxRα, and ABCA-1 expression in HUVEC exposed to uremia

**Fig. 2:**
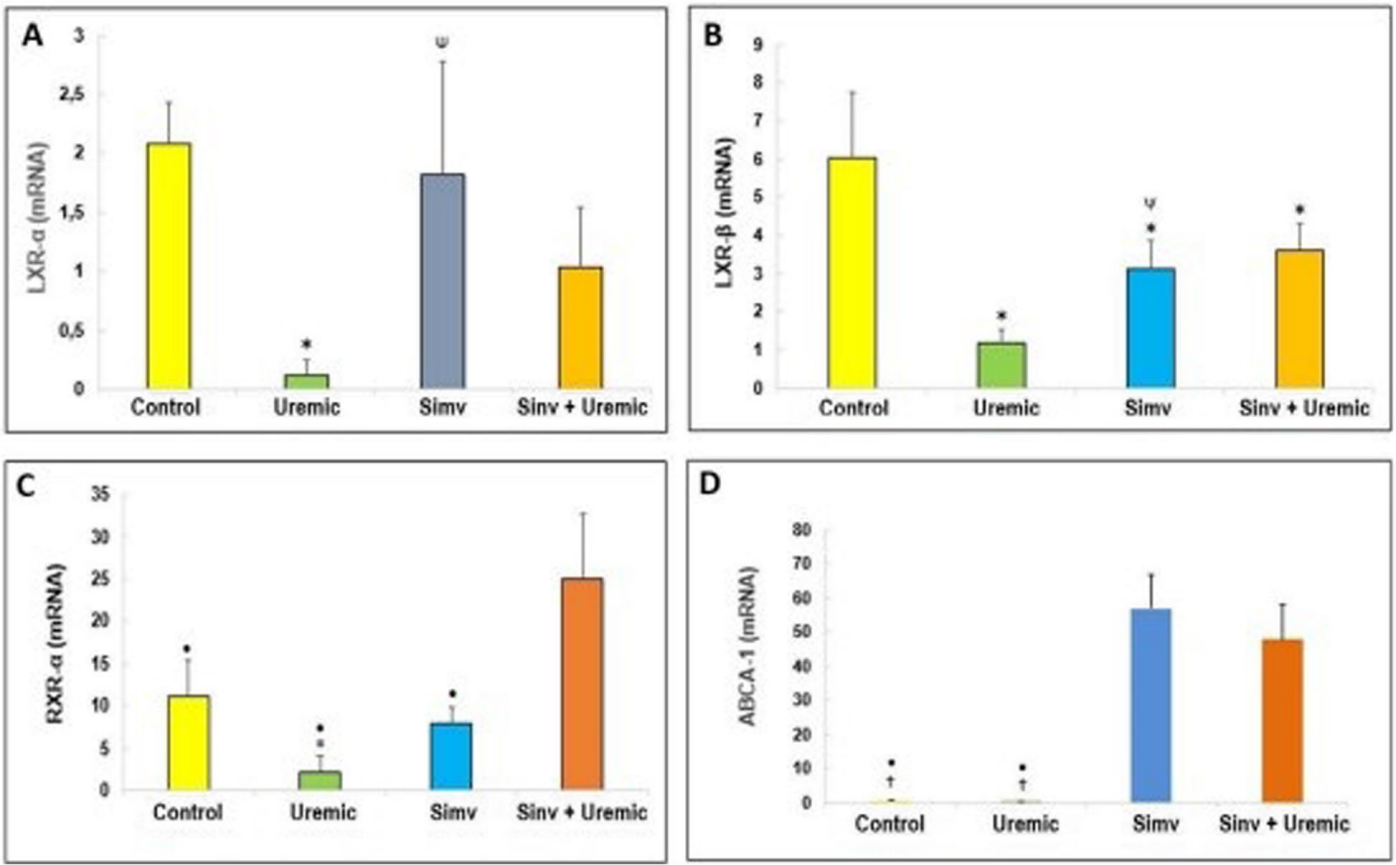
Expression (relative mRNA, normalized to HPRT) of: **A**) LXR-α, **B**) LXR-β, **C**) RXR-α and **D **ABCA-1 in HUVEC incubated with serum from control, uremic serum, pretreated with simvastatin and pretreated and subjected to insult with uremic serum for 24 hours. *p≤0.05 versus control; ^Ψ^versus Uremic; ^†^*p*≤0.05 versus Simvastatin; ^◾^*versus *Simvastatin + Uremic (*N*=12)

We found that the uremic environment reduces the expression of LXRα compared to cells incubated with serum from healthy individuals (relative mRNA: 0.11 ± 0.14 *versus* 2.08 ± 0.34, *P* < *0.05*). Pretreatment with simvastatin increased LXRα expression in HUVEC compared to cells incubated with uremic serum (relative mRNA: 1.82 ± 0.95 *versus* 0.11 ± 0.14, *P* < *0.05*). Pre-incubation with simvastatin reversed the decrease observed in LXRα expression when cells were exposed to uremia (relative mRNA: 1.03 ± 0.51 *versus* 0.11 ± 0.14, *P* < *0.05*) (Fig. 2a).

LXRβ expression was significantly higher in cells incubated with serum from healthy individuals compared to cells from the other groups studied (relative mRNA: control: 6.05 ± 1.73 *versus* uremic: 1.20 ± 0.77; simvastatin: 3.13 ± 0.33; simvastatin + uremic: 3.61 ± 0.73, *P* < *0.05*). Pre-incubation with simvastatin for 24 h reversed the decrease in LXRβ expression observed in cells incubated with uremic serum (relative mRNA: simvastatin + uremic: 3.61 ± 0.73 *versus* 1.20 ± 0.77, *P* < *0.05*) (Fig. 2b).

The uremic environment decreased RXRα expression compared to the group of cells exposed to the serum of healthy individuals (2.05 ± 2.10 *versus* 11.16 ± 4.14, *P* < *0.05*). There was a statistically significant increase in RXRα expression in simvastatin-pretreated cells incubated with uremic serum compared to the other groups (relative mRNA: simvastatin + uremic: 24.90 ± 7.81 *versus* healthy: 11.16 ± 4.14; uremic: 2.05 ± 2.10; simvastatin: 7.86 ± 1.96, *P* < *0.05*) (Fig. 1c).

The cells pretreated with simvastatin had increased expression of ABCA-1 compared to the healthy and uremic groups (relative mRNA: simvastatin: 57.81 ± 10.50 *versus* control: 0.69 ± 0.19 and uremic: 0.08 ± 0.07, *P* < *0.05*).

Simvastatin increased ABCA-1 expression in cells only incubated with the statin compared with the control and uremic groups. This increase was observed even when the pretreated HUVEC we re exposed to uremic serum compared to the group exposed to serum from healthy individuals (relative mRNA: 48.43 ± 10.51 *versus* 0.69 ± 0.19, *P* < *0.05*) and the group exposed to uremic serum (relative mRNA: 48.43 ± 10.51 *versus* 0.08 ± 0.07, *P* < *0.05*) (Fig. 1d).

### The receptor LxRβ correlates directly with LxRα and ABCA-1, and inversely with TNF-α

**Fig. 3:**
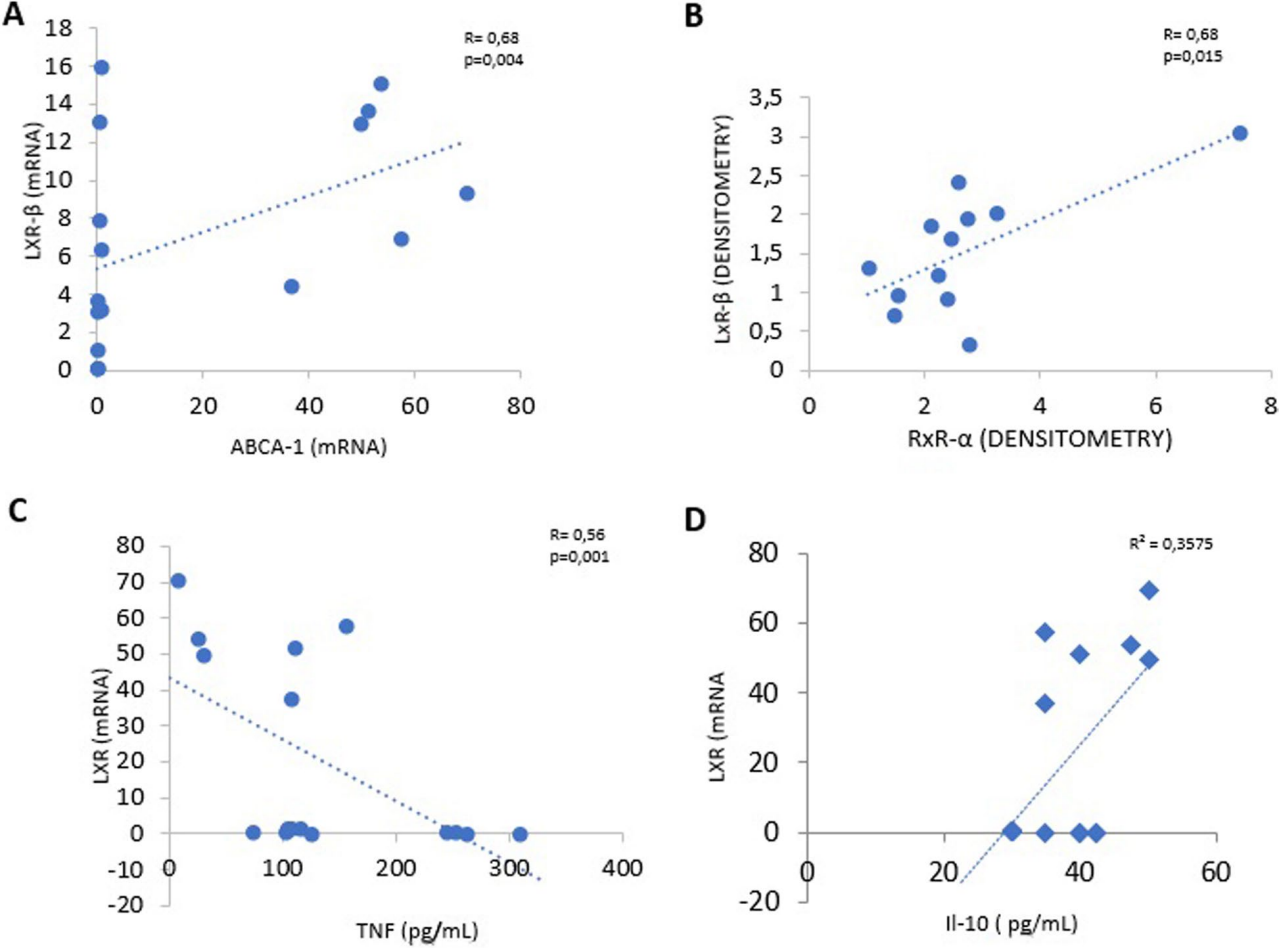
Spearman’s rank correlation analysis showing the correlation between: **A** LXR-β versus ABCA-1 expression, **B**, LXR- β versus RXRa, C TNF-α versus ABCA-1 expression, and **D** IL-10 versus ABCA-1 expression

LxRβ expression showed a direct correlation with ABCA-1 expression (r: 0.68; *p* < *0.004*) and with RXRα (R: 0.68; *P* < *0.015*) (Fig. 3A and B).

TNF-α secretion correlated inversely with LxRβ (R: −0.56; *p* < *0.006*). No correlation was observed with IL-10 secretion (Fig. 3D).

### Simvastatin increases LxR-β, RXRα, and ABCA-1 promoter activities in HUVEC

**Fig. 4:**
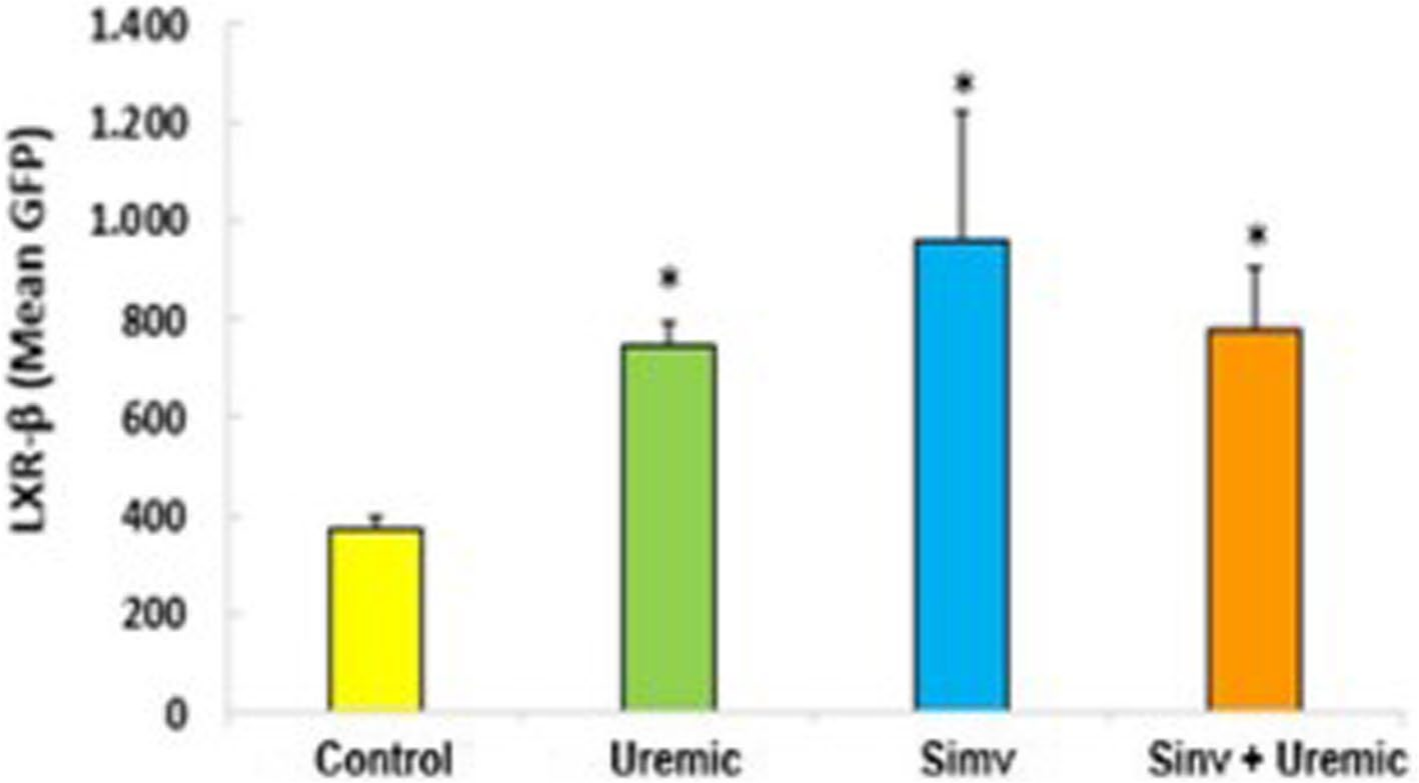
Activation of LxR-β transcription in HUVEC cells incubated with serum from control, uremic serum, pretreated with simvastatin and pretreated and subjected to insult with uremic serum for 24 hours. *p*≤0.05 (^*^versus control), *N*=6

We examined the upstream regulatory regions by transfecting HUVECs with LxR, RXRα, and ABCA1 promoter-driven reporter constructs. As shown in Fig. 4, the activation of the transcription sequence of the LxR-β receptor in HUVEC was higher in all groups compared to control (control: 373.20 ± 23.02 *versus* uremic: 743.20 ± 53.50; simvastatin: 956.67 ± 266.00; simvastatin + uremic: 781.33 ± 123.00, *P* < *0.05*).

**Fig. 5:**
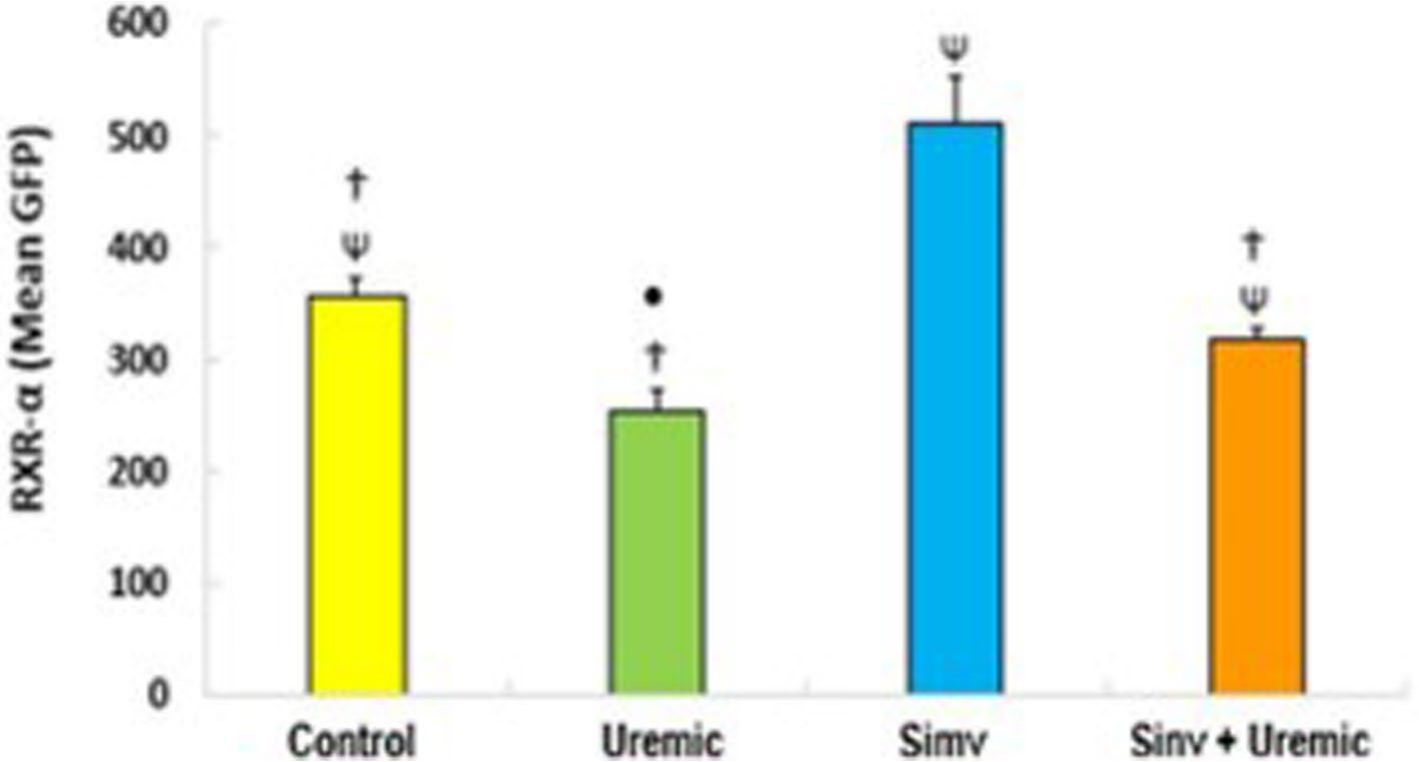
Activation of RXR-α transcription in HUVEC cells incubated with serum from control, uremic serum, pretreated with simvastatin and pretreated and subjected to insult with uremic serum for 24 hours. p≤0.05 (^*^versus control; ^Ψ^versus uremic; ^†^versus simvastatin; ^• +^ Uremic), *N*=6

In Fig. 5, we observed increased activation of RXRα transcription when comparing the control group with cells incubated with simvastatin (357.31 ± 15.32 versus 511.47 ± 41.35, *P* < *0.05*). Cells incubated with uremic serum showed decreased RXRα activities that were reversed with concomitant incubation with simvastatin (uremic: 254.40 ± 18.30 *versus* simvastatin + uremic: 317.03 ± 10.17, *P* < *0.05*).

**Fig. 6:**
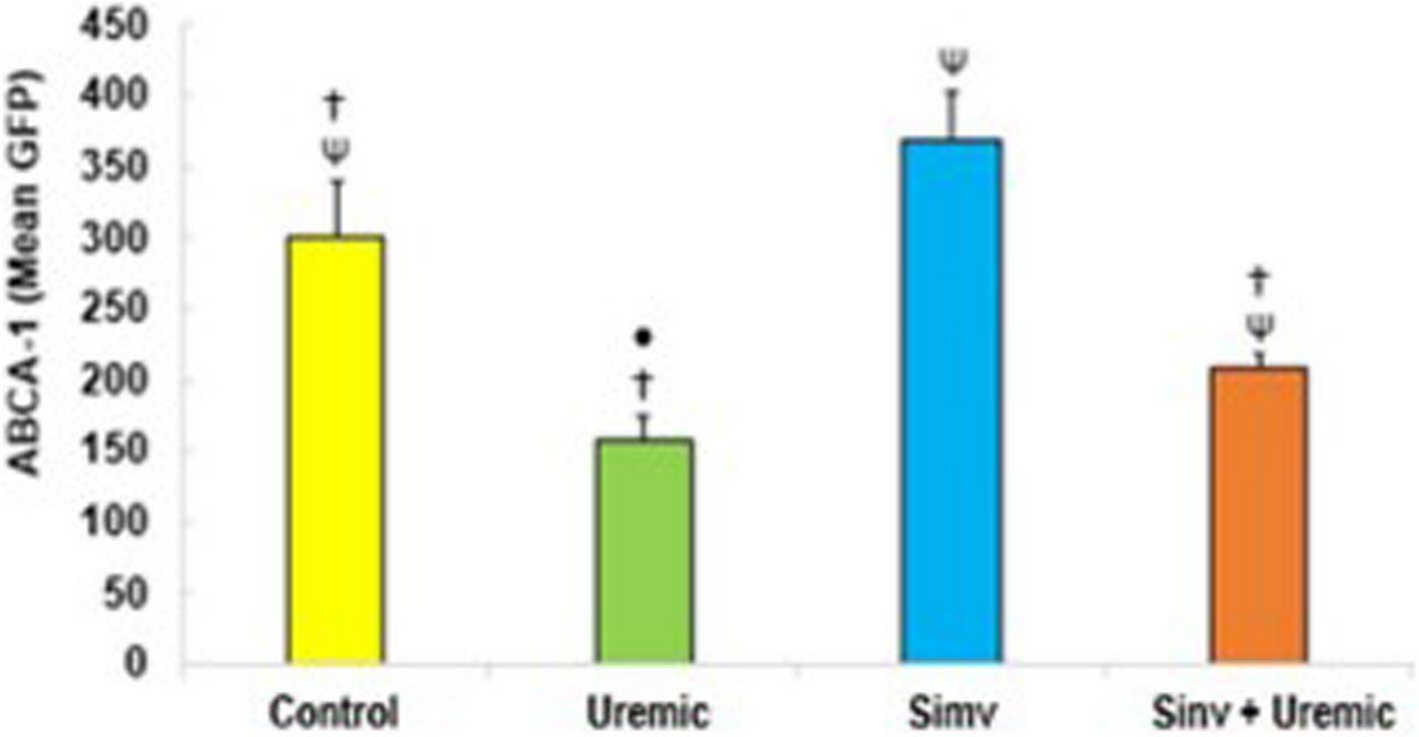
Activation of ABCA-1 transcription in HUVEC cells incubated with serum from control, uremic serum, pretreated with simvastatin and pretreated and subjected to insult with uremic serum for 24 hours. *p*≤0.05 (^*^versus control; ^Ψ^versus uremic; ^†^versus simvastatin; ^•^versus Simvastatin + Uremic), *N*=6

The uremic environment inhibited the activation of ABCA-1 transcription compared to the other groups (uremic: 158.60 ± 16.04 *versus* control: 301.67 ± 38.60; simvastatin: 369.60 ± 32.62; simvastatin + uremic: 207.60 ± 10.50, *P* < *0.05*). The transcription activation of the receptor sequence was higher in the group pretreated with simvastatin compared to the other groups (simvastatin: 369.60 ± 32.62 *versus* control: 301.67 ± 38.60; uremic: 158.60 ± 16.04; simvastatin + uremic: 207.60 ± 10.50, *P* < *0.05*). In HUVEC exposed to uremia and pretreated with simvastatin, we found increased activation compared to the uremic group (207.60 ± 10.50 *versus* 158.60 ± 16.04, *P* < *0.05*) (Fig. 6).

**Fig. 7:**
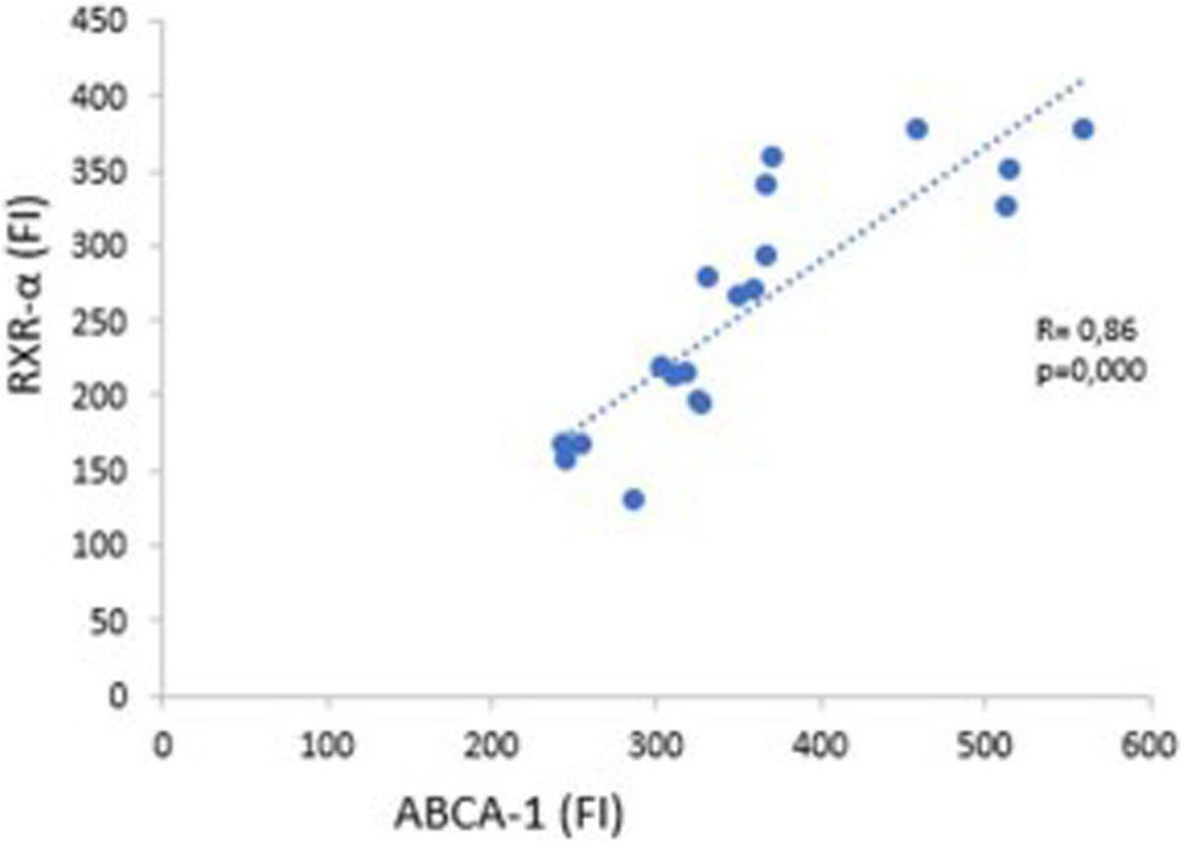
Correlation between RXR-α expression and ABCA-1

In the correlation coefficient analysis, the activation of RXRα transcription was directly correlated with that of ABCA-1 (R: 0.86; *P* < *0.001*) (Fig. 7).

### Diminished protein expressions of LxRβ, RXRα, and ABCA1 exposed to uremia were attenuated in pretreatment with simvastatin

**Fig. 8:**
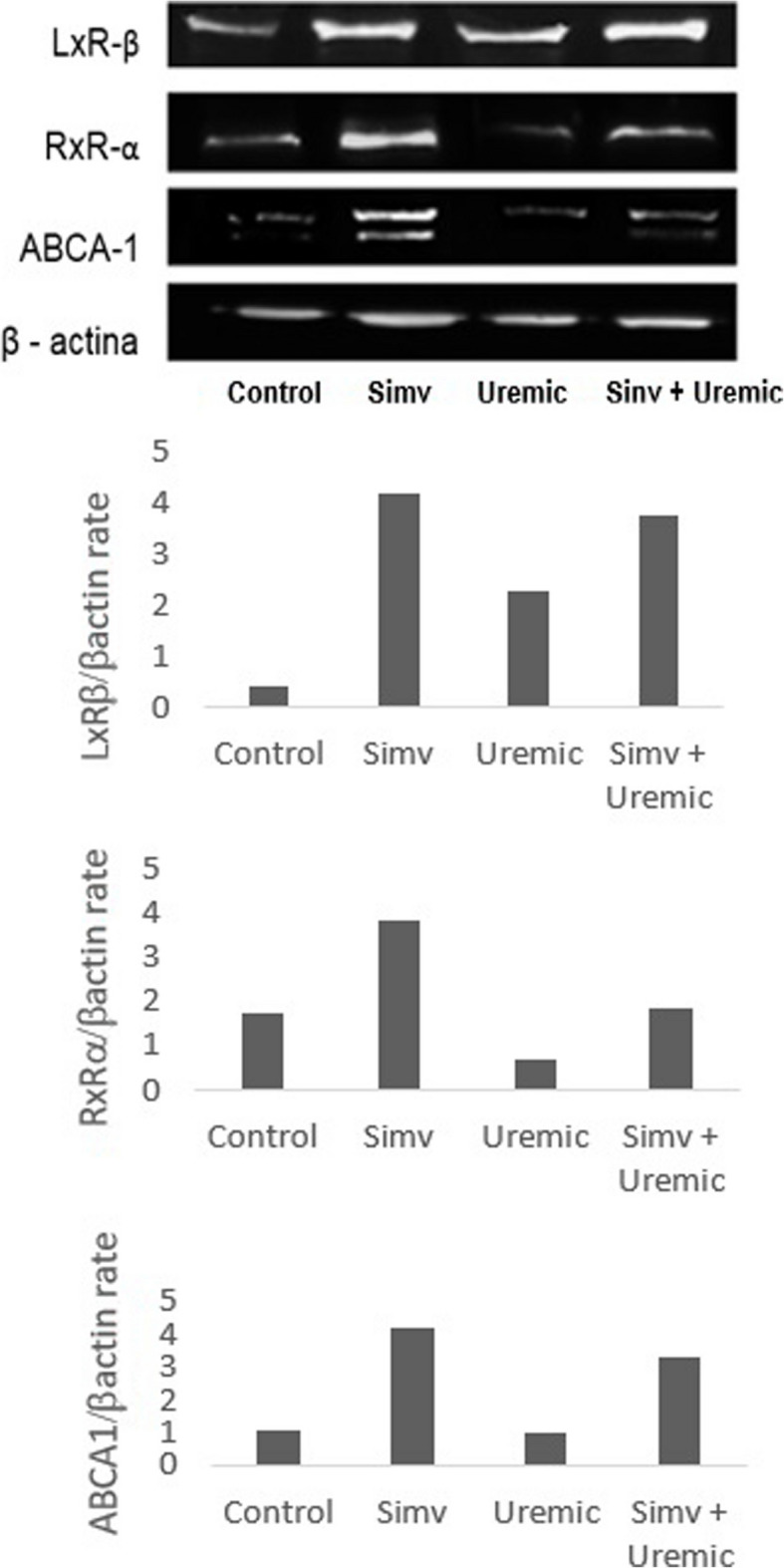
Analysis of protein expression of LXR-β, RXR-α, and ABCA-1 transporters in HUVECs incubated with serum from control, uremic serum, simvastatin-pretreated serum, and serum pretreated with simvastatin and subjected to an insult with uremic serum for 24 hours. The protein expression levels are shown as the ratio of each protein expression levels are shown as the ratio of each protein to β-actin and proteins bands densities were quantified using imageJ software and are represented in bar graphs below the western blot image

As observed in Fig. 8, protein expression of LxRβ, RXRα, and ABCA1 was higher when cells were treated with simvastatin. Additionally, compared to the group of HUVEC exposed to uremia, cells concomitantly pretreated with the statin presented increased expression of all analyzed proteins.

## Discussion

This study investigates the interaction between the uremic environment and endothelial inflammatory responses, focusing on the pivotal roles of the cytokines TNF-α and IL-10, their effects on the expression of LXR and RXR receptors, and the impact of statins, specifically simvastatin, in modulating these responses. The results demonstrated that the uremic environment significantly increased TNF-α secretion by HUVEC cells, reinforcing the critical role of this cytokine in vascular lesion formation. In contrast, IL-10 secretion was lower in endothelial cells exposed to serum from healthy individuals, whereas uremic serum stimulated an increase in this anti-inflammatory cytokine. Moreover, we observed that pretreatment with simvastatin, before exposure to the uremic environment, reduced TNF-α secretion and enhanced ABCA-1 expression, highlighting its beneficial effects in modulating inflammatory responses and endothelial protection [24–26]. In contrast, it was observed that IL-10 secretion by endothelial cells was lower in those incubated with serum from healthy individuals compared to HUVEC exposed to uremic serum. IL-10 is an anti-inflammatory cytokine commonly secreted by immune cells such as leukocytes and lymphocytes Indeed, a delay in IL-10 secretion occurs when stimulated by pro-inflammatory factors [24]. In uremia, endotoxins and complement activation contribute to increased IL-10 secretion by macrophages and monocytes [24]. Additionally, TNF-α stimulates the increase of IL-10 levels through feedback, negatively regulating the secretion of this pro-inflammatory protein [24]. The clearance of IL-10 is renal, and its half-life is significantly increased in the plasma of patients with CKD. However, this increase attenuates adhesion molecules and leukocyte recruitment by reducing the release of chemotactic substances [24], indicating that its action is potentially protective in the atherosclerotic process, although the mechanisms by which it interacts with different molecules are not entirely elucidated.

In the present study, it was also investigated the possible mechanism involving the anti-inflammatory role of ABCA-1 in endothelial cells. The heterodimers formed by RXR activation by LXR induce the transcription and expression of ABC family transporters, particularly ABCA-1. ABCA-1 transporters regulate cholesterol transport and increase high-density lipoprotein (HDL) [25], playing a crucial role in the removal of excess cholesterol from peripheral tissues to the liver, leading to biliary excretion and characterizing reverse cholesterol transport [27].

Here, it was demonstrated that the uremic environment significantly reduces the expression of LXRα, LXRβ, and RXRα compared to expression in healthy individuals. In the inflammatory scenario, cytokines such as TNF-α, IL-1β, and IFN-γ negatively regulate the transcription of ABCA-1 mediated by LXR, as well as the expression of the protein [28], reinforcing the complex interplay between cholesterol response manipulation and inflammatory responses. Studies have shown that the activation of LXR has a global anti-inflammatory effect on macrophages, linked to the coordinated activation of multiple genes [28]. In fact, our findings were reinforced by the inverse correlation observed between TNF-α secretion and receptor expression.

It was observed that the uremic environment significantly reduced the expression of LXRα, LXRβ, and RXRα, which was primarily at the transcriptional level, as evidenced by decreased mRNA levels. In contrast, the increase in ABCA-1 expression observed in cells pretreated with simvastatin was associated with both transcriptional and post-translational regulation, as it involved increased mRNA expression and subsequent enhancement in protein levels, as indicated by the changes in receptor and transporter protein expression. The results also suggest that decreased gene expression of LXR and RXRα receptors in the uremic environment was associated with diminished expression of ABCA-1. It was observed a decrease in ABCA-1 expression and transcription activity in the uremic environment compared to the group pretreated with simvastatin and the group that was pretreated and incubated with uremic serum. HDL-mediated RCT is an atheroprotective biological mechanism impaired during inflammatory states [25]. A study in human hepatoma cells showed that the acute inflammatory response decreases the effectiveness of cholesterol efflux in macrophages [25]. Baranova et al. demonstrated that treatment with lipopolysaccharide in murine macrophages causes downregulation in the expression of ABCA-1 and SR-B1 receptors, leading to consequent inhibition of cholesterol efflux compared to expression in untreated cells [29].

The increase in LXRβ expression in the group of cells with serum from healthy individuals was significant compared to the other groups, just as LXRα expression under the same conditions when compared to the uremic group. When observed individually, LXR receptors can be activated in different scenarios, such as dyslipidemia. The involvement of specific ligands for each homologous receptor results in the activation of several intracellular signaling pathways [30], leading to increased transcription of the receptor and protein expression in inflammatory environments, as demonstrated in this study.

The potential benefits of statins were also explored independently of their primary function of reducing circulating cholesterol levels. These effects are attributed to their pleiotropic actions, promoting anti-inflammatory, antioxidant effects, and vascular bed protection [31, 32]. In animal models, this finding was confirmed with statin administration, where renoprotection was observed with the preservation of glomerular function, preventing chronic kidney disease [26]. In clinical practice, the reduction of dyslipidemia was demonstrated with the use of pravastatin in patients with CKD, resulting in decreased disease progression and attenuation of cardiovascular burden [33].

Pretreatment with 10 µM of simvastatin for 24 h reduced TNF-α secretion and increased IL-10 levels in HUVEC cells, even when exposed to the uremic environment, indicating that statins play an important role in mitigating inflammatory cytokine secretion. Indeed, a previous study demonstrated the effects of statins modulated by TNF-α in 3T3-L1 differentiated adipocytes [34].

It was also demonstrated that cells pretreated with simvastatin showed increased ABCA-1 expression compared to the healthy and uremic groups, highlighting the beneficial effects of 3-hydroxy-3-methylglutaryl-CoA reductase inhibitors in modulating LXRβ and RXRα receptors and, consequently, in increasing ABCA-1 transcription. ABCA-1 transcription increased significantly in cells incubated with simvastatin compared to the uremic group. The group pretreated with simvastatin and exposed to uremia also showed a significant increase in transcription compared to the uremic group, corroborating protein mRNA expression.

Additionally, simvastatin increased LXRβ and RXRα transcription, suggesting that the drug positively modulates gene transcription of these receptors. In line with this, simvastatin treatment resulted in increased protein expression of LXRβ, RXRα, and the ABCA-1 transporter, indicating positive post-translational modulation in cells exposed to the drug. Changes in a cell's lipid concentration due to statin activity can act on endogenous ligands and nuclear receptor modifiers. Moreover, statins are close homologues of the lipid metabolites they inhibit and, therefore, can function as nuclear receptor ligands [35].

### Strengths and Limitations

#### Strengths

This study investigates the effects of statins on ABCA1 expression in endothelial cells specifically under uremic conditions, an area that has been relatively underexplored. The use of endothelial cells exposed to uremic serum allows for controlled experimental conditions, facilitating a clearer understanding of the mechanisms involved. The study not only evaluates ABCA1 expression but also explores the anti-inflammatory effects of statins, providing a comprehensive view of their potential vascular protective roles. By linking laboratory findings to potential clinical outcomes, the research addresses a significant gap in understanding the cardiovascular implications for patients with chronic kidney disease.

#### Limitations

While in vitro studies provide valuable insights, they may not fully replicate the complexities of human physiology and the multifactorial nature of uremia. The study may not account for long-term effects and outcomes of statin treatment in CKD patients, limiting the applicability of findings to clinical practice. If the study utilizes a small number of endothelial cell lines or conditions, the generalizability of results may be restricted. Finally, while the study examines ABCA1 and inflammatory pathways, other relevant mechanisms influencing cardiovascular health in CKD may not be explored.

## Conclusion

In conclusion, it was demonstrated that the uremic environment reduces the expression of LXRα, LXRβ, and RXRα, leading to a consequent decrease in ABCA-1 expression in HUVEC cells. Simvastatin reversed this deleterious effect of the uremic milieu, indicating a beneficial action of statins in modulating LXRβ and RXRα, ultimately promoting increased expression and transcription of ABCA-1 receptors and improving RCT. Additionally, the anti-inflammatory potential of simvastatin was evident, as it mitigated the secretion of pro-inflammatory cytokines such as TNF-α, further supporting its protective role in vascular health. However, further studies are needed to elucidate whether such specific transcriptional modulation of RXRα, LXRβ receptors, and the ABCA-1 transporter can be translated into tangible clinical benefits, particularly in inflammatory diseases such as CKD.

## Supplementary Information

Supplementary File 1.

## Data Availability

No datasets were generated or analysed during the current study.

## Declarations

### Data sharing statement

The data supporting the findings of this study are not available in a public repository. However, the data are available upon request from the corresponding author.
