# Supplementary material for: Action of 3-Hydroxy-3-Methylglutaryl-CoA Reductase Inhibitors on ABCA-1 protein (ATP-Binding Cassette Transporter-1) in endothelial cells stimulated with uremic serum
Source: Lipids Health Dis. 2025 Mar 19;24:100. doi: 10.1186/s12944-024-02420-6 (PMC11921485; doi:10.1186/s12944-024-02420-6)
Supplement: Supplementary file 1 [file 12944_2024_2420_MOESM1_ESM.pdf]

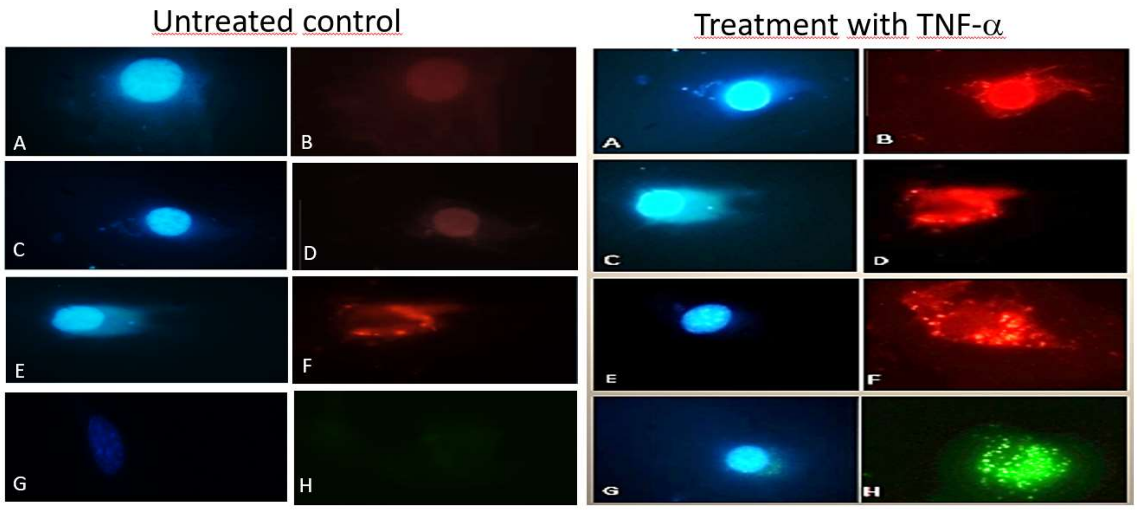

Fig.1: Characterization of HUVEC cells by immunofluorescence. A, C, E, and G: Anti-nuclear labeling using DAPI; B: Constitutive marker CD146; D: Constitutive marker CD31; F: Cells exposed to 100 ng/mL of TNF-α for 2 hours and labeled with anti-VCAM; H: Constitutive marker Von Willebrand factor (vWF).

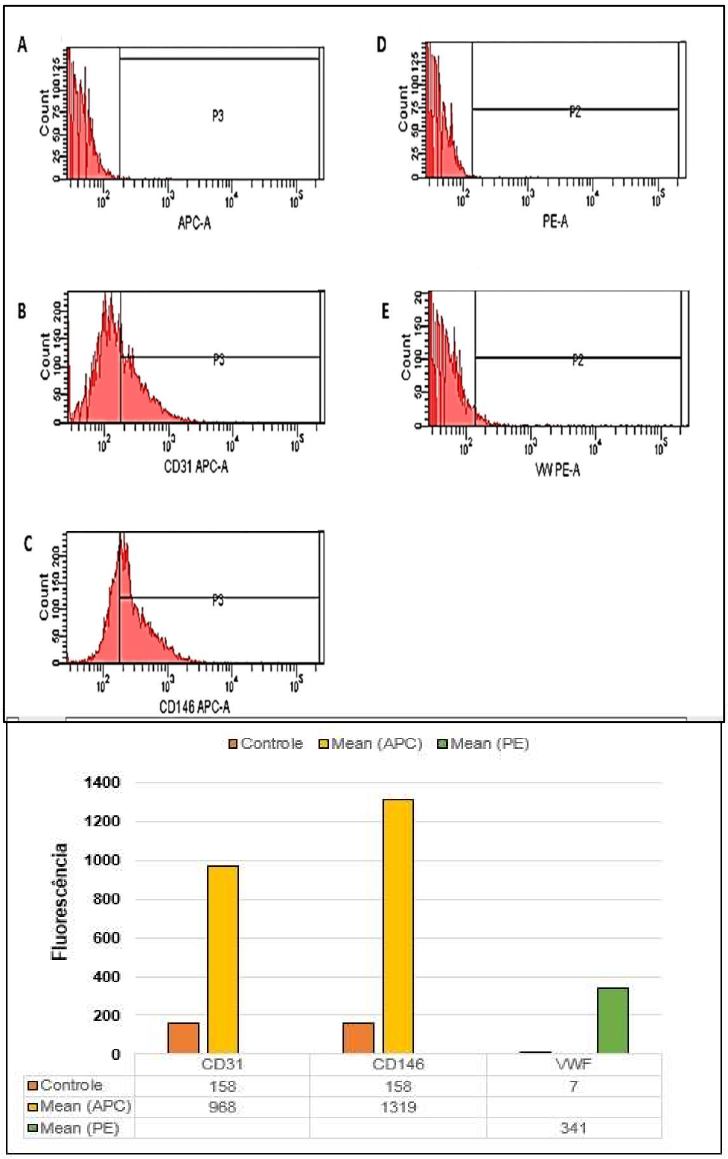

	CD31	CD146	VWF
Controle	158	158	7
Mean (APC)	968	1319	
Mean (PE)			341

Fig.2: Characterization of HUVEC cells by flow cytometry. Above: A. Negative control: HUVECs labeled with the APC fluorophore; B. HUVECs labeled with anti-CD31 antibody conjugated to APC; C. HUVECs labeled with anti-CD146 antibody conjugated to APC; D. Negative control: HUVECs labeled with the PE fluorophore; E. HUVECs labeled with anti-vWF antibody conjugated to PE. These graphs were obtained from flow cytometry analysis. Below: Expression of CD31, CD146, and vWF, as constitutive markers for HUVEC characterization, expressed as the mean fluorescence intensity (MFI).

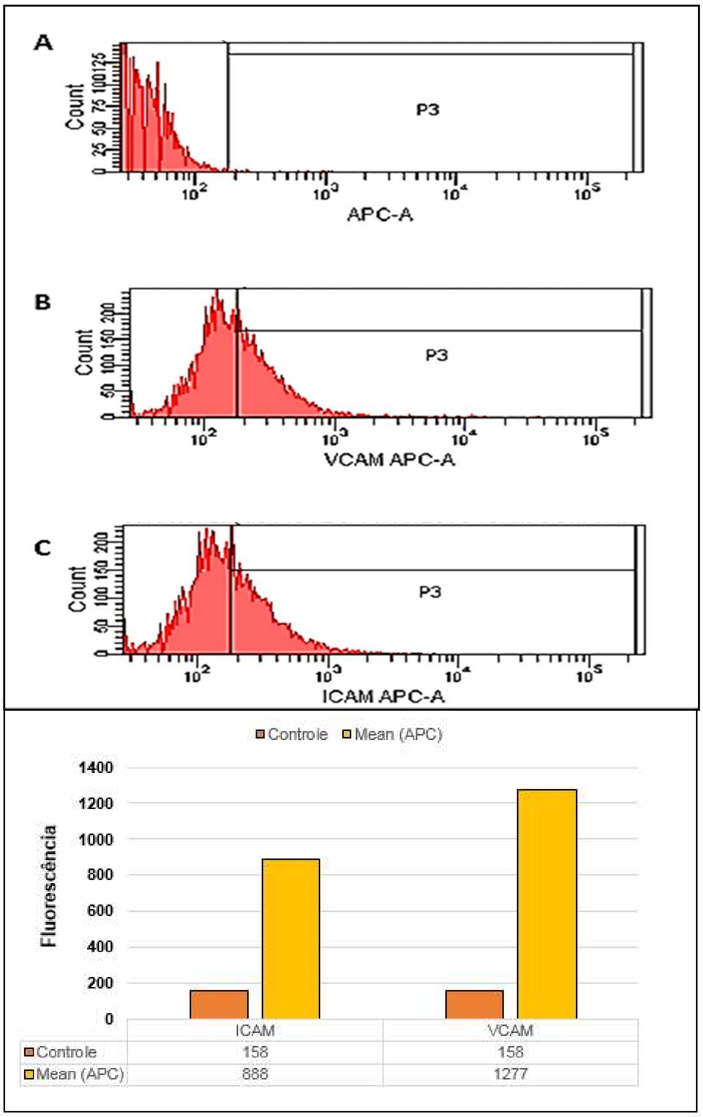

Fig.3: Characterization of HUVEC cells by flow cytometry. Above: A. Control: HUVECs labeled with the APC fluorophore; B. HUVEC cells labeled with anti-VCAM antibody conjugated to APC; C. HUVEC cells labeled with anti-ICAM antibody conjugated to APC. Panels B and C show cells exposed to 100 ng/mL of TNF-α for 2 hours. Below: Expression of VCAM and ICAM as specific markers for HUVEC characterization, expressed as the mean fluorescence intensity (MFI). The graphs were obtained from flow cytometry analysis of HUVEC cells.
